# A behavioral and molecular study; ameliorated anxiety‐like behavior and cognitive dysfunction in a rat model of chronic unpredictable stress treated with oregano extract

**DOI:** 10.1002/brb3.2727

**Published:** 2022-07-27

**Authors:** Parvaneh Mohseni‐Moghaddam, Manijeh Dogani, Motahare Hatami, Samira Roohollahi, Azam Amiresmaeli, Nayereh Askari

**Affiliations:** ^1^ Department of Physiology, Faculty of Medicine, Tehran Medical Sciences Islamic Azad University Tehran Iran; ^2^ Department of Biology, Faculty of Sciences Shahid Bahonar University of Kerman Kerman Iran; ^3^ Department of Cardiology, Tehran Heart Center Tehran University of Medical Sciences Tehran Iran; ^4^ Immunoregulation Research Center Shahed University Tehran Iran

**Keywords:** anxiety‐like behavior, behavioral tests, chronic unpredictable stress, cognitive dysfunction, gene expression, *Origanum vulgare* L., toxicity assessment

## Abstract

**Objective:**

Chronic stress is considered a severe risk factor leading to various disorders, including anxiety and cognitive decline. The present study aimed to investigate the effects of *Origanum vulgare* (oregano) extract on improving anxiety‐like behavior and learning and memory defection caused by chronic unpredictable stress (CUS).

**Method:**

A 10‐day CUS protocol was executed on male rats, and on day 10, their anxiety, learning, and memory status were evaluated. After that, in addition to the CUS, the rats were treated with the oregano extract for 2 weeks. Then, the expression of BDNF, TrkB, and TLR2/4 genes in the hippocampus and prefrontal cortex of the rats was evaluated. Also, the liver‐ and kidney‐related serum parameters, including triglycerides, total cholesterol, HDL, LDL, creatinine, urea, serum glucose, alanine aminotransferase, and aspartate aminotransferase were assessed. Further, the extract's lethal effect and its impact on animals’ body weight were investigated.

**Results:**

Behavioral tests confirmed the anxiety‐like behavior and learning–memory function impairment caused by CUS. In contrast, the administration of the extract could significantly alleviate the mental deficiencies and diminished anxiety‐like behaviors. Molecular assessments showed that CUS could markedly decrease the BDNF and TrkB genes’ expression levels while increasing that of TLR2 and TLR4. In contrast, in extract‐treated animals, mRNA levels of BDNF and TrkB considerably increased, yet TLR2 and TLR4 mRNA levels reduced. Additionally, consumption of the extract caused weight gain, while having no lethality and detrimental effect on the liver and kidneys functions.

**Conclusions:**

These findings indicate the anxiolytic properties of the extract and its improving effect on cognitive dysfunction.

## Graphical abstract

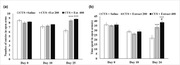

## 1 INTRODUCTION

Today, due to increasing exposure to social challenges, the majority of individuals suffer from chronic stress as a crucial mental health issue. Chronic stress is commonly known as a principal risk factor causing various mental disorders, including anxiety, depression, and cognition‐related impairments, such as learning and memory deterioration (Meynaghizadeh‐Zargar et al., 2020). It has been clarified that the hippocampus and prefrontal cortex brain regions are involved in anxiety‐related behaviors, in addition to learning and memory formation (Lam et al., 2018; Li et al., 2017; Liu et al., 2014; Okoh et al., 2020). There is solid evidence that these two critical areas are affected by chronic stress (Okoh et al., 2020). Studies have shown that stress affects the cellular integration and active involvement of these brain regions (Mousavi et al., 2020). In the same way, multiple investigations have demonstrated that the neuroinflammation caused by stress increases the production of oxygen radicals inducing adverse effects in the hippocampus and killing neurons (Blossom et al., 2020; Cerqueira et al., 2007).

At the molecular level, memory decline and anxiety‐like behavior are associated with an alteration in the expression of several genes, including brain‐derived neurotrophic factor (BDNF), tyrosine kinase‐coupled receptor (TrkB), and toll‐like receptors 2 and 4 (TLR2/4) (Amiresmaeili et al., 2018; Bondi et al., 2008; Cieśelik et al., 2011). Among the neurotrophins, BDNF and its major receptor, TrkB, have the most expression in the brain of the developing and adult mammalian. They are involved in the differentiation and survival of neuronal cells (Cunha et al., 2010). Hence, the deficiency of BDNF expression or the signaling pathway triggered by TrkB is linked to cognitive impairment and the development of anxiety‐like behavior (Ji et al., 2017; Kemppainen et al., 2012; Liu et al., 2014).

The involvement of TLR2 and TLR4 mediated signaling pathways in the development of neurodegenerative diseases has recently been revealed (Kwilasz et al., 2021; Zhou et al., 2020). TLR2 and TLR4 are a class of pattern recognition receptors (PRRs) that initiate the innate neuroinflammatory response and have been identified in neurons and glial cells, such as astrocytes and microglia (Mao et al., 2012). Activation of these receptors results in the up‐regulation of activator protein‐1 (AP‐1) and nuclear factor‐kappa B (NF‐κB), stimulating the synthesis and secretion of pro‐inflammatory cytokines (Zhou et al., 2020). Furthermore, it has been revealed that TLR2 and TLR4‐associated signaling pathways are involved in cognitive dysfunction and anxiety behavior by inducing neuroinflammation (Kwilasz et al., 2021; Nie et al., 2018; Zhou et al., 2020). Besides, a recent study carried out by our group revealed that the mRNA level of TLR2 and TLR4 increased in the hippocampal tissue and prefrontal cortex of animals subjected to stress (Amiresmaeili et al., 2018). It is hypothesized that some antioxidant and anti‐inflammatory agents could reduce TLR2/4 levels and control the stress consequences.

Plants are rich in natural bioactive compounds and are a potential source of natural antioxidants, including phenolic compounds, flavonoids, alkaloids, and tannins (Dogani et al., 2021; Khorrami et al., 2019). In this regard, *Origanum vulgare* L., also known as oregano or marjoram, is an aromatic plant belonging to the Lamiaceae family widely distributed throughout Asia, especially in Iran (Amiresmaeili et al., 2018). Previous studies have demonstrated that the extracts of marjoram/oregano have antioxidant, anti‐cancer, antimicrobial, and anti‐inflammatory activities (Arcila‐Lozano et al., 2004). Recently, an in vivo study has demonstrated the antidepressant and anxiolytic activities of oregano extract (Mechan et al., 2011). Likewise, the anxiolytic properties of thymol and carvacrol, two main components of the oregano extract, have been reported in several studies (Kaewwongse et al., 2013; Melo et al., 2010; Pezzani et al., 2017). Previous studies have also revealed that oregano extract consumption can improve learning and memory in rats (Ghaderi et al., 2020; Sheibani et al., 2011). Additionally, in traditional medicine, this plant is used to alleviate respiratory disorders, stomach upsets, and indigestion. These properties are mainly attributed to the bioactive components, such as γ‐terpinene, p‐cymene, 4‐terpineol, thymol, trans‐sabinene hydrate, carvacrol, β‐caryophyllene, and β‐caryophyllene oxide, which are abundantly present in this plant extract (Pezzani et al., 2017). However, to the best of our knowledge, the effectiveness of this herbal extract on anxiety and cognitive deficits induced by chronic unpredictable stress (CUS) has not been investigated yet.

Herein, we designed an experiment to study the potential of oregano extract to improve learning and memory impairment induced by stress. We also investigated the possible effects of this treatment on the expression of genes involved in the dysfunctions. For this purpose, the effect of two concentrations of the oregano extract on the performances of the animals subjected to CUS was examined. At the end of the treatment period, the expression of TLR2, TLR 4, BDNF, and its receptor TrkB genes in the animals were evaluated. Also, the extract's impact on animals’ body weight, blood biochemical parameters, and mortality rate (at higher doses) was investigated.

## 2 EXPERIMENTAL

### 2.1 Animals

The male Wistar rats (190‐240 g) were purchased from the Laboratory Animal Center of Kerman University of Medical Sciences and housed under 12:12 light/dark cycles and ad libitum access to water and food. Before the beginning of the experiments, the animals were allowed to acclimatize to these new conditions for 7 days. All the animal protocols applied in the present research followed the animal ethics committee guidelines of the Kerman Neuroscience Research Center (EC/KNRC/89‐5A).

### 2.2 Oregano extract preparation

The aerial parts of the oregano plant were provided in the early summer of 2019 from local markets of the city of Yazd (Yazd Province, Iran) and authenticated by Dr. Seyed Mansour Mirtadzadini, a botanist at the Shahid Bahonar University of Kerman, Kerman, Iran. Then, the plant leaves were dried in the shadow, powdered, and their extract was prepared based on the maceration method. Briefly, 100 g of the obtained powder was mixed with 1 L distilled water and shaken for 3 days at 30˚C. After that, the mixture was filtered using a Whatman filter paper (Grade 1), concentrated via a rotary evaporator at 45°C, and dried at room temperature.

### 2.3 Experimental design

The animals were randomly assigned to one of the following 6 groups of treatment (8 rats per group).

1. Extract 200: the rats daily received 200 mg/kg of the oregano extract (solved in 0.5 ml of saline) for 14 days.
2. Extract 400: the rats daily received 400 mg/kg of the oregano extract (solved in 0.5 ml of saline) for 14 days.
3. Vehicle group: the rats daily received 0.5 ml of saline for 14 days.
4. CUS+ Extract 200: the rats were subjected to CUS for 24 days. From the 10th day onward, along with the CUS, the rats received 200 mg/kg of the oregano extract (solved in 0.5 ml of saline) for 14 days.
5. CUS+ Extract 400: the rats were subjected to CUS for 24 days. From the 10th day onward, along with the CUS, the rats daily received 400 mg/kg of the oregano extract (solved in 0.5 ml of saline) for 14 days.
6. CUS+ saline: the rats were subjected to CUS for 24 days. From the 10th day onward, along with the CUS, the rats daily received 0.5 ml of saline for 14 days.

**FIGURE 1:**
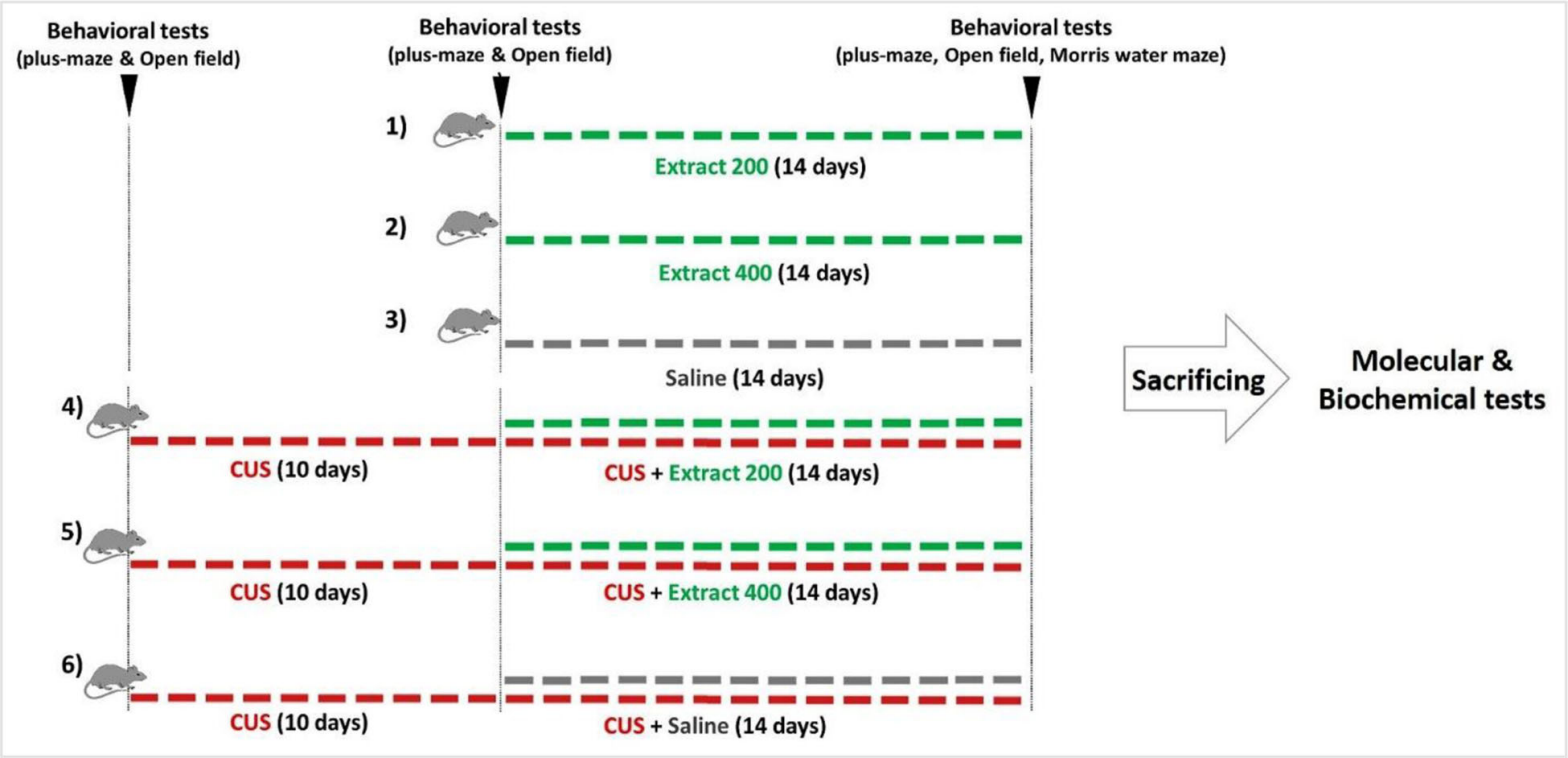
Study experimental design. This diagram shows how each group of rats was treated and when the behavioral and molecular tests were performed.

Here, the saline and/or the extract treatment was done by oral administration. Also, behavioral tests related to anxiety, including EPM and open field, were performed on days 0, 10, and 25 during CUS induction. Morris water maze test was also conducted at the end of the CUS protocol to evaluate the spatial learning and memory of the animals. Further, the rats’ weights were measured on the first and last days of the experiment. Figure 1 illustrates all 6 groups of the investigation.

### 2.4 Chronic unpredictable stress (CUS) protocol

Chronic unpredictable stress (CUS) provides a well‐accepted animal social stress model leading to memory decline and anxiety‐like behavior (Bondi et al., 2008). During this process, different stressors with a diverse sequence were applied each week to prevent any habituation. Animals were subjected to two different types of stress each day for 24 days. Details of the CUS protocol performed in this study are presented in Table 1 (Ma et al., 2013).

**TABLE 1:** Protocol used for chronic stress induction for 24 days

Day	1	2	3	4	5	6	7	8
Stressor	15 min forced swim (20°C), 1 min tail pinch	12 h cage tilting (45°C), 1 h cage rotation	reversal of the light/dark cycle, 1 h cold room (4°C)	12 h wet bedding, crowded cage	24 h food deprivation, 1 h restraint	12 h cage tilting (45°C), crowded cage	24 h water deprivation, 1 h cold room isolation	reversal of the light/dark cycle, 1 min tail pinch
Day	9	10	11	12	13	14	15	16
Stressor	cold room (4°C), 1 h cage rotation	24 h water and food deprivation, 12 h cage tilting(45°C)	15 min forced swim (20°C), 1 h restraint	reversal of the light/dark cycle, 24 h food deprivation	1 min tail pinch, cold room (4°C)	24 h water deprivation, 1 h restraint	12 h wet bedding, 12 h cage tilting (45°C)	1 h cage rotation, reversal of the light/dark cycle
Day	17	18	19	20	21	22	23	24
Stressor	1 h restraint, crowded cage	12 h wet bedding, 1 min tail pinch	reversal of the light/dark cycle, 12 h cage tilting (45°C)	15 min forced swim (20°C), 24 h water Deprivation	1 h cage rotation, crowded cage	24 h food deprivation, 1 min tail pinch	1 h restraint, 12 h wet bedding	24 h water and food deprivation, crowded cage

**Definitions**: Restraint: animals were housed in a restrainer made of plexiglass and flexible nylon, which restricted movement but did not affect respiration and air circulation. Tail pinch: it means placing the animal in the previously described restrainer and applying a clothespin approximately 1 cm from the base of the tail. Crowded cage: 8 rats per cage.

### 2.5 Behavioral tests

#### 2.5.1 Elevated plus‐maze test

One of the behavioral tests used to assess anxiety‐related behaviors in rats was the EPM test. The EPM test was performed before stress induction (day 0), on the tenth day of the CUS protocol, and after the last day of the CUS protocol (day 25). In summary, the EPM apparatus was made of wood and consisted of four arms, including two open arms (50 × 10 cm) and two closed arms (50 × 10 cm), which extended from a central platform (10 × 10 cm) and elevated 50 cm above the floor. The maze apparatus was located in a quiet room, and the light intensity on the device was 40–55 lux. Each animal was placed in the center of the platform facing the open arm and searched the EPM for 5 min. Also, a video tracking system installed on the top and center of the maze was used to track animals’ behavior. The number of times each animal entered the open arm and the time spent in this arm were recorded.

#### 2.5.2 Open‐field test

The open‐field test was carried out in a wooden square arena with the dimensions of 70 × 70 × 30 cm. The animals (8/group) were permitted to freely investigate the field for 5 min. The time spent, as well as the number of entries of the animals into the center of the box (covering 40 × 40 cm), was recorded by a video camera. The open‐field test, like the EPM test, was performed on days 0, 10, and 25.

#### 2.5.3 Morris water maze (MWM)

The MWM behavioral test was conducted after the CUS induction. A black circular tank with a diameter of 150 cm and a wall of 75 cm in height was filled with tap water (21 ± 2˚C). A platform made of Plexiglas was embedded 1 cm below the water level. The black tank was settled in a room with several visual cues on the walls surrounding the circular pool. A video camera that was installed on top of the tank recorded the total duration and the distance each rat traveled to find the hidden platform during the training phase. In addition, the number of entries into the target quadrant, the time spent in the quadrant, and the distance traveled during the retention phase were recorded. According to the protocol, the learning trials (training phase) included four blocks (4 trials per block), with a 5‐min rest between the blocks. A trial was started by putting the animal in the tank in one of the four randomly selected directions. In each trial, the animal was permitted to swim for 1 min to find the concealed platform. Whenever the rat could not find the hidden platform within 1 min, it was removed from the water and gently guided to the platform, where it remained for 15 s between trials. Finally, on the probe day (remembering phase), which was conducted 24 h after the last block, the platform was taken out from the pool, and the animals were allowed to search it for 1 min (Mohamadi‐Jorjafki et al., 2020; Mohseni‐Moghaddam et al., 2019).

At the end of the behavioral tests, all animals were sacrificed, and their blood was collected to evaluate blood biochemical parameters.

### 2.6 Molecular analysis

#### 2.6.1 RNA extraction and cDNA synthesis

The animal hippocamp and prefrontal cortex tissues were removed to assess BDNF, TrkB, and TLR2/4 genes expression. Total RNA was extracted from these tissues by the RNX‐Plus reagent (CinnaGen Co., Iran). The purified RNA was dissolved in 30 μl of DEPC‐treated water and analyzed via a NanoDrop™ 2000/2000c spectrophotometer. Also, the integrity of the extracted RNA was confirmed by 1.5% agarose gel (Sigma, Taufkirchen, Germany) electrophoresis. Afterward, cDNA synthesis was conducted with oligo‐dT primer and M‐MuLV reverse transcriptase (Fermentas kit, St. Leon‐Rot, Germany), according to the manufacturer's instructions.

#### 2.6.2 Real‐time polymerase chain reaction (RT‐PCR)

The cDNA prepared from each sample was applied to evaluate the mRNA expression of BDNF, TrkB, TLR2, and TLR4 genes using a Bio‐Rad iQ5 detection system (Bio‐Rad, Richmond, USA). This was carried out using Real Q Plus 2× Master Mix (Ampliqon, Denmark). Also, glyceraldehyde 3‐phosphate dehydrogenase gene (*gapdh*) has been used as an internal control for normalizing the expression of target genes. The real‐time PCR program used in this study was as follows: initial denaturation at 95°C for 15 min followed by 40 cycles for real‐time PCR, including 20 s at 95°C (denaturation phase), 30 s at 60°C (annealing phase), and 30 s at 72°C (elongation phase). A final melting curve was performed to check for product specificity and primer dimers. For further confirmation, products from each primer pair were also loaded to 1.5% agarose gel electrophoresis. Primer sequences, RT‐PCR product length, and their NCBI accession number are shown in Table 2. Noteworthy, all samples were evaluated in duplicate, and their means were used in subsequent analysis. The relative mRNA levels were calculated by the delta CT method.

**TABLE 2:** Details of materials used in the RT‐PCR procedure

Primer name	Primer sequence	PCR product size	NCBI accession number
GAPDH	F: GTCTTCACCACCACGGAGAAGGC	392	NM‐017008
	R: ATGCCAGTGAGCTTCCCGTTCAGC		
BDNF	F: CGTGATCGAGGAGCTGTTGG	343	XM‐008762078
	R: CTGCTTCAGTTGGCCTTTCG		
TrkB	F: TGACGCAGTCGCAGATGCTG	245	NM‐012731
	R: TTTCCTGTACATGATGCTCTCTG		
TLR2	F: GGGTTCTGACATTGGAGTCC	182	XM‐008761102/1
	R: CAGTGTCCTGTAAGGATTTCC		
TLR4	F: CGGAAAGTTATTGTGGTGGTGT	121	NM‐019178/1
	R: GGACAATGAAGATGATGCCAGA		

### 2.7 Assessment of the serum levels of biochemical parameters

To evaluate the effect of the extract (400 mg/kg) on blood biochemical parameters, after anesthetizing, the animals’ blood was taken by cardiac puncture and centrifuged to separate serum. Next, different parameters, namely triglycerides (TG), total cholesterol, HDL, LDL, creatinine, urea, serum glucose, alanine aminotransferase (ALT), and aspartate aminotransferase (AST), were analyzed using relevant standard kits (KONELAB 20XT, Finland) according to the manufacturer's instructions.

### 2.8 Lethal effect of the extract

In order to evaluate the rats’ mortality rate under treatment with high concentrations of the extract, 16 healthy rats were divided into 4 groups and orally administered one of the following doses of the extract, 800, 1600, 3200, and 4000 mg/kg, once a day for 3 days. The animals were monitored for a week to assess their mortality rate.

### 2.9 Statistical analyses

Data analysis was performed using SPSS 23.0 (IBM, Chicago, IL, USA). All the results are reported as mean value ± SEM. One‐way analysis of variance (ANOVA) followed by the Tukey's post‐hoc test was done to evaluate statistical differences between different groups.

## 3 RESULTS

### 3.1 The influence of the extract administration on anxiety‐like behavior

#### 3.1.1 The EPM test

**FIGURE 2:**
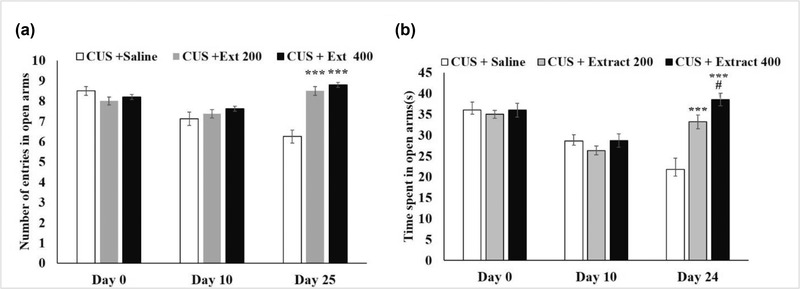
Effects of CUS and the extract (200 mg/kg and 400 mg/kg) on the time animals spent in the open arms **(a)** and the number of entries into the open arms **(b)**. Each point represents the mean value ± SEM ^***^ *p* < 0.001 versus Day 0; ^#^ *p* < 0.05, versus Day 10

As demonstrated in Figure 2a,b, during this 25‐day procedure, the number of times that CUS‐subjected rats entered the open arms and the time they spent there continually decreased. In contrast, these features saw a considerable increase in the groups treated with the extract. Nearly no differences were observed between the animals' behaviors on day 25 and day 0. Notably, the concentration of 400 mg/kg of the extract showed to be slightly more effective than 200 mg/kg.

#### 3.1.2 The open‐field test

**FIGURE 3:**
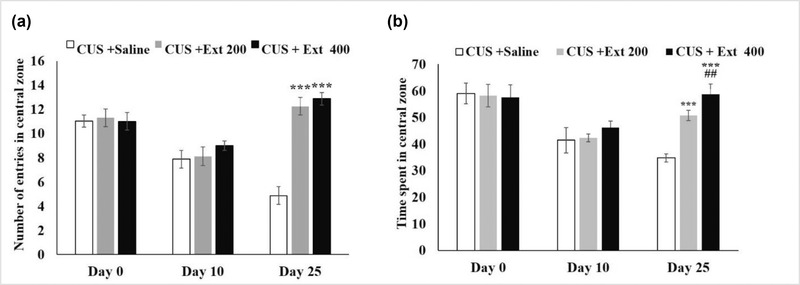
Effects of CUS and the extract (200 and 400 mg/kg) on the number of entries of the rats into the central zone **(a)** and time spent there **(b)**. Each point represents the mean value ± SEM. ^***^ *p* < 0.001 compared to Day 0; ^###^ *p* < 0.01 versus Day 10. CUS: chronic unpredictable stress; Extract: *Origanum vulgare* L. extract

Results of the open‐field test revealed that the experience of CUS for 24 days significantly influenced animal behaviors. The number of entrances into the central zone and the time CUS‐induced rats spent in this area reduced continuously. These figures declined from 11 times and 60 s on day 0 to 5 times and 35 s on day 25, respectively. This is when the consumption of both concentrations of the extract (200 and 400 mg/kg) since the tenth day of the test could considerably improve these features. The rats that received 400 mg/kg of the extract, for example, entered the central zone an average of 12 times and spent about 60 s there on day 25. And no marked difference was observed between the performance of this group and the control (Figure 3).

#### 3.1.3 Morris water maze test (MWM)

The MWM test was conducted to evaluate the spatial learning and memory impairments of the animals with CUS‐induced anxiety. Based on the results (Table 3), anxiety caused a significant increase in the total duration and distance the rats traveled to find the hidden platform in the training phase. However, these parameters diminished when the animals were treated with the extract (200 and 400 mg/kg). Also, the induction of chronic stress led the rats to be less likely to enter the target quadrant, less distance was traveled there, and less time spent in the area compared to the vehicle group on the probe test. However, oral administration of the oregano extracts significantly relieved the impact of stress on the animals’ behaviors. The extract treatment at the doses of 200 and 400 mg/kg could increase the number of entries, distance traveled, and the time that rats spent in the target quadrant. Noteworthy, the rats that only received 400 mg/kg of the extract showed the best performance, followed by the rats subjected to CUS, and 200 or 400 mg/kg of the extract.

**TABLE 3:** Effects of administration of 200 and 400 mg/kg of the extract on spatial learning and memory deficits in animals that were subjected to CUS protocol

	Performance					
	**Learning**			**Memory**		
Treatments	Duration (s)	Distance (m)	Speed (m/s)	Number of entries	Times (s)	Distance (m)
Vehicle	21 ± 1	1 ± 0.1	0.058 ± 0.001	6.8	22 ± 0.8	3.1 ± 0.3
Extract 200	15 ± 1**	1.8 ± 0.1*	0.068 ± 0.002	9**	26.8 ± 0.9*	3.9 ± 0.1*
Extract 400	14 ± 1**	0.96 ± 0.1*	0.069 ± 0.001	10***	32.3 ± 1.5***	5.1 ± 0.3***
CUS + Saline	31 ± 2***	2.2 ± 0.1***	0.072 ± 0.003	4.1***	11.8 ± 0.5***	1.6 ± 0.1***
CUS + extract 200	25 ± 2^##^	1.8 ± 0.2** ^##^	0.072 ± 0.004	7.4^###^	20 ± 0.9^###^	3 ± 0.5^###^
CUS + extract 400	21 ± 1^##^	1 ± 0.1^###^ ^$$^	0.068 ± 0.002	8.2^###^	27.2 ± 0.4^###^ ^$$^	4.1 ± 0.2^###^ ^$$^

n = 8 rats/group; .

^^ *p* < 0.01, and.

^^^ *p* < 0.001 in comparison with the CUS group.

* *p* < 0.05.

** *p* < 0.01 and.

*** *p* < 0.001 compared to vehicle group.

^$$^ *p* < 0.01 versus CUS+ extract 200.

CUS: chronic unpredictable stress; Extract: *Origanum vulgare* L. extract.

### 3.2 Gene expression (*BDNF, TrkB, TLR2*, and *TLR4*)

**FIGURE 4:**
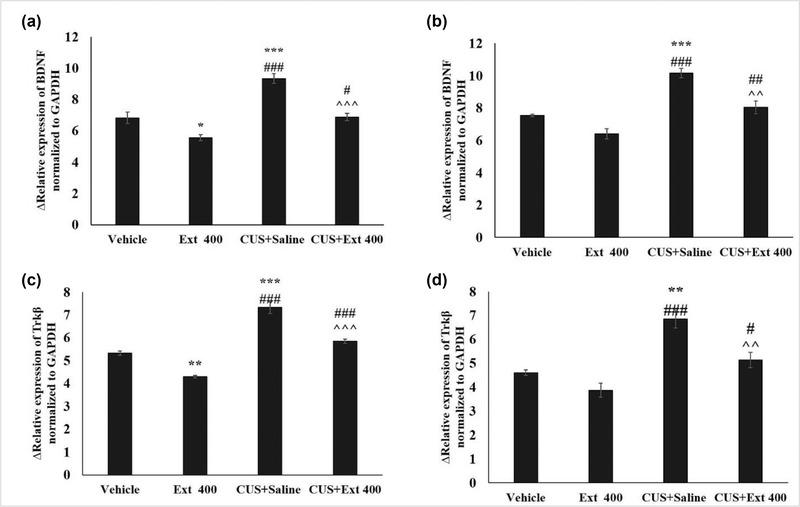
Effects of CUS and treatment with extract (400 mg/kg) on the gene expression of BDNF, TrkB, TLR2, and TLR4 in the hippocampal tissue (a and c) and the prefrontal cortex (b and d). Each point represents the mean ± SEM. ^*^ *p* < 0.05, ^***^ *p* < 0.001 in comparison with the vehicle group, ^#^ *p*< 0.05, ^##^ *p*< 0.01, and ^###^ *p* < 0.001 versus extract 400 group, ^^*p* < 0.01 and ^^^*p* < 0.001 compared to CUS+Saline group. CUS: chronic unpredictable stress; Extract: *Origanum vulgare* L. extract

**FIGURE 5:**
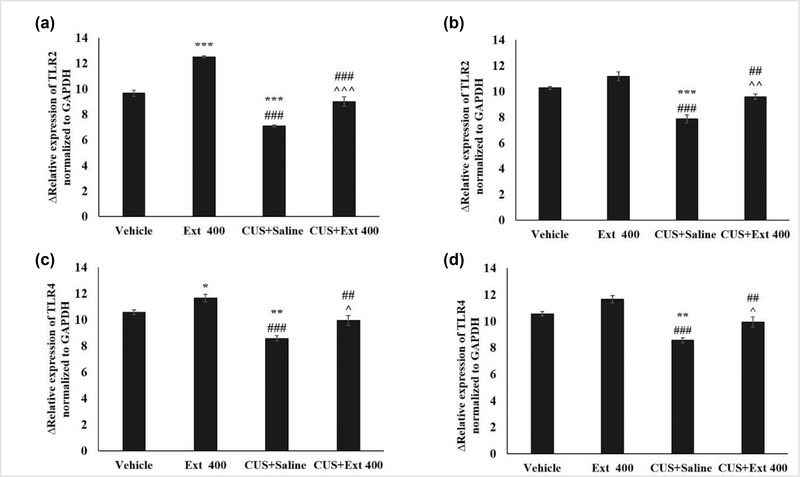
Effects of CUS and the extract treatment (400 mg/kg) on TLR2 and TLR4 mRNA expression in the hippocampal tissue (a and c) and the prefrontal cortex (b and d). Each point represents the mean value ± SEM. ^***^ *p* < 0.001 compared to vehicle group, ^##^ *p* < 0.01 and ^###^ *p* < 0.001 versus OVE 400 group, ^^*p* < 0.01 and ^^^*p* < 0.001 in comparison with the CUS+Saline group. CUS: chronic unpredictable stress; Extract: *Origanum vulgare* L. extract

As shown in Figures 4 and 5, the expression of BDNF and TrkB in both prefrontal and hippocampal tissues of the group experiencing CUS was significantly reduced in comparison to the vehicle group. The extract administration, in contrast, attenuated this effect, and treatment of stressed animals with the extract at the dose of 400 mg/kg statistically increased BDNF and TrkB mRNA expression in prefrontal and hippocampal tissues, in comparison with the CUS group. The obtained results also showed that TLR2 and TLR4 mRNA levels in the prefrontal and hippocampal tissues of the animals of the CUS group were significantly higher than in the vehicle group. However, this rise reversed after administering the extract. Our findings indicate that utilization of 400 mg/kg of the extract could reduce the mRNA expression of TLR2 and TLR4 in stressed rats.

### 3.3 Serum levels of biochemical parameters

The obtained results show that stress induction in animals could significantly elevate AST, ALT, urea, serum glucose, LDL, and cholesterol levels but reduce HDL level compared to the vehicle group. However, creatinine and triglyceride levels were not affected in the stressed animals. On the other hand, treating the stressed animals with the extract (400 mg/kg) for 14 consecutive days significantly decreased serum glucose levels, ALT, AST, urea, and LDL compared to the group under CUS. Also, the extract treatment increased the serum levels of HDL so that, as indicated in Table 4, no significant difference in HDL level was observed between CUS + extract and vehicle groups.

**TABLE 4:** Effect of the extract on serum biochemical factors

Factors	Treatments			
	Vehicle	Extract 400 (g/kg)	CUS + Saline	CUS + Extract 400 (g/kg)
AST (IU/L)	192 ± 9	166 ± 6	284 ± 3** ^##^	229 ± 4** ^###^ ^^^
ALT (IU/L)	96.4 ± 3.9	90 ± 2.6	168 ± 2*** ^###^	121 ± 2*** ^###^ ^^^
Urea (mg/dl)	34.6 ± 1.5	25 ± 1**	53 ± 2*** ^###^	41 ± 0.7^###^ ^^^
Creatin (mg/dl)	0.38 ± 0.01	0.36 ± 0.02	0.46 ± .02	0.38 ± .02
Glucose (mg/dl)	101.8 ± 2.4	93 ± 3	149 ± 5*** ^###^	117 ± 2* ^##^ ^^^
LDL (mg/dl)	24.4 ± 0.6	25 ± 1	34 ± 0.6*** ^###^	28 ± 0.6*** ^###^ ^^^
HDL (mg/dl)	55 ± 2	49 ± 1	45 ± 2*	48 ± 3
TG (mg/dl)	65 ± 4	62 ± 1	69 ± 2	63 ± 1
Cholesterol (mg/dl)	53.4 ± 1.3	52 ± 1	69 ± 3** ^##^	64 ± 2* ^##^

Values are given as mean ± SEM.

* *p* < 0.05.

** *p* < 0.01, and.

*** *p* < 0.001 in comparison with the vehicle group.

^##^ *p* < 0.01 and.

^###^ *p* < 0.001 versus the extract group (400 g/kg).

^^^ *p* < 0.001 compared to CUS+Saline group.

CUS: chronic unpredictable stress; extract: *Origanum vulgare* L. extract.

### 3.4 Bodyweight

Before starting the experiment (day 0), there were no statistical differences in animal weights among experimental groups. Yet, at the end of the treatment, the bodyweight of the stressed animals markedly decreased in comparison with the vehicle group. After administration of the extract at both doses (200 and 400 mg/kg), this stress‐induced weight loss was significantly attenuated (Table 5).

**TABLE 5:** The effect of the extract/CUS treatments on rats’ body weight

Groups	Initial weight (g)	Final weight (g)	**Bodyweight difference (g)**
Vehicle	196 ± 2	245 ± 2^^^	49
Extract 200 g/kg	197 ± 2	243 ± 3^^^	46
Extract 400 g/kg	201 ± 2	243 ± 2^^^	42
CUS + Saline	195 ± 2	198 ± 1	3
CUS + extract 200 g/kg	193 ± 1	209 ± 2^, ^###^ ^&&&^	14
CUS + extract 400 g/kg	193 ± 3	209 ± 2^ ^###^ ^&&&^	14

Values are given as mean ± SEM.

^ *p* < 0.05 and.

^^^ *p* < 0.001 versus CUS + saline group.

^###^ *p* < 0.001 versus OVE 400.

^&&&^ *p* < 0.001 compared to Extract 200.

CUS: chronic unpredictable stress; Extract: *Origanum vulgare* L. extract.

### 3.5 The lethal effect of the extract

Based on the obtained results, no lethal effect was observed by acute administration of the extract even at high concentrations of 800, 1600, 3200, and 4000 mg/kg during the 72‐h follow‐up period.

## 4 DISCUSSION

Long‐term exposure to stress is a key risk factor that causes anxiety and cognitive deficits (Meynaghizadeh‐Zargar et al., 2020). Results of the present study indicate that anxiety‐like behavior induced by stress was significantly alleviated by consuming the oregano extract. As previous animal studies showed, thymol, one of the main components found in the oregano, could substantially reduce anxiety. The proposed mechanism of action is that this compound induces its effect through an agonistic/modulatory activity on the gamma‐aminobutyric acid receptor (GABA_A_R) (Bhandari & Kabra, 2014; Kaewwongse et al., 2013). Moreover, the anxiolytic effect of carvacrol, another major component of oregano extract, has been reported already. Based on investigations, the anxiolytic effect of this component is limited in the presence of flumazenil, a selective GABA_A_ receptor antagonist, indicating its probable mechanism of action via the GABA_A_ receptor (Melo et al., 2010; Pezzani et al., 2017). Cognitive impairment is another disorder related to stress. In this study, our data demonstrated that consumption of the oregano extract significantly improved spatial learning and memory deterioration induced by stress. Likewise, studies have revealed that oregano extract consumption improves learning and memory in normal rats, possibly owing to its antioxidant activity and inhibition of acetylcholinesterase (Ghaderi et al., 2020; Sheibani et al., 2011). In addition, the essential oil extracted from oregano has been shown to ameliorate cognitive dysfunction in Alzheimer's rats (Postu et al., 2020).

Different molecular signaling pathways, such as BDNF, TrkB, TLR2, and TLR4, are involved in the pathogenesis of anxiety and cognitive decline (Fereidooni et al., 2020; Liu et al., 2014; Mao et al., 2012). Based on the results, BDNF and TrkB mRNA levels in animals subjected to CUS combined with receiving the extract increased, and the TLR2/4 mRNA levels decreased in these animals’ hippocampal tissue and prefrontal cortex. Studies have demonstrated that BDNF is a critical factor for the development and function of the 5‐HT neurotransmitter, which is a key modulatory neurotransmitter linked to anxiety and depression. Besides, it has been reported that BDNF is required for the anxiolytic and antidepressant actions of selective serotonin reuptake inhibitors (SSRI), which are the first line of pharmacological interventions for anxiety and depression treatment (Duman, 2017). Generally, due to the effect of this essential neurotrophic factor on survival, synaptic plasticity, and release of neurotransmitters, its decreased expression appears to cause destructive effects on many mental and behavioral activities. This is when the oregano extract, through increasing the expression of BDNF and its receptor, could have a protective effect against stress‐related disorders.

TLR2 and TLR4 have been identified as crucial mediators in generating neuroinflammation, leading to neuronal changes and anxiety behavior. Inhibition of TLR2 and TLR4, though, can abolish stress‐induced anxiety (Nie et al., 2018). Besides, it has been reported that TLRs activation results in altered synaptic activity, leading to altered behaviors in animals (Chen et al., 2019). Arguably, oregano extract via attenuating TLR2/4 expression can diminish neuro‐inflammation. So, it can be a potentially therapeutic substance to reduce anxiety and ameliorate learning and memory impairments caused by chronic stress.

The aim of the present study concerning blood biochemical parameters was to investigate the effect of the consumption of the extract on the function of liver and kidney organs. Based on the results, this extract not only did not adversely affect the mentioned organs but also reduced the detrimental effects of the stress on biochemical factors. As mentioned in the literature, CUS by affecting liver function and the adrenal gland profoundly affects lipid and glucose metabolism and increases the level of LDL, triglyceride, cholesterol, and blood glucose, while decreasing HDL levels. Moreover, hepatic enzymes, including AST and ALT, as quantitative markers of liver injury, also increase following stress induction (Jia et al., 2016; Nayanatara et al., 2009). According to previous studies, CUS‐treated animals showed elevated levels of urea and creatinine, indicating kidney function disturbance and severe glomerular and tubular damage (Bin‐Jaliah, 2016). It is also well characterized that stressful events lead to weight loss (Jia et al., 2016). All of the quoted points were well in agreement with our results.

The exact mechanism involving the extract in ameliorating the psycho‐behavioral and biochemical effects of stress is still unclear. The reduction of blood glucose by the extract may be relevant to its mitigating effect on stress (Dinardo et al., 2020). In another study, the ameliorating effect of oregano hydroalcoholic extract on serum biochemical factors has been attributed to its anti‐inflammatory and anti‐oxidant characteristics (Sharifi‐Rigi et al., 2019). Also, the observed weight gain in the extract‐treated rats can be related to the appetite enhancer feature of the origanum (Allan & Bilkei, 2005). It is worth mentioning that in too‐high doses, the oregano oil has shown to be toxic and even lethal. This may be attributed in part to the presence of thymol and carvacrol, two of the monoterpene phenols it contains. In high doses, these components are irritants that can affect the skin or internal organs, and induce detrimental effects (Borgarello et al., 2015; Tisserand & Young, 2014). In contrast, this extract did not show any lethality even at high concentrations, making it an invaluable agent to relieve anxiety.

## 5 CONCLUSION

The obtained results from the present study demonstrated that CUS leads to the incidence of behavioral disorders and memory impairment. Also, it was revealed that stress could decline the expression of BDNF and TrkB genes while increasing the expression of TLR2 and TLR4. The consumption of the oregano extract, on the other hand, could alleviate anxiety‐like behaviors and ameliorate learning and memory impairment. This extract up‐regulated the expression of BDNF and TrkB genes and down‐regulated TLR2 and TLR4 genes’ expression in the prefrontal and hippocampus areas. Possibly, in this way, it could prevent mental disorders caused by stress.

## CONFLICT OF INTEREST

The authors declare no conflict of interest.

## AUTHOR CONTRIBUTIONS

**Parvaneh Mohseni‐Moghaddam: c**ontributed to the experimental part of the study, data analysis/interpretation, and drafting of the manuscript. **Manijeh Dogani**: contributed to the experimental part of the study, data analysis, interpretation, and revising. **Motahare Hatami**: participated in the data analyzing and revising of the manuscript. **Samira Roohollahi** and **Azam Amiresmaeli**: contributed to the experimental part of the study. **Nayereh Askari**: supervised the work, provided the materials, and contributed to the conception or design of the work

### PEER REVIEW

The peer review history for this article is available at https://publons.com/publon/10.1002/brb3.2727.
